# htsget: a protocol for securely streaming genomic data

**DOI:** 10.1093/bioinformatics/bty492

**Published:** 2018-06-19

**Authors:** Jerome Kelleher, Mike Lin, C H Albach, Ewan Birney, Robert Davies, Marina Gourtovaia, David Glazer, Cristina Y Gonzalez, David K Jackson, Aaron Kemp, John Marshall, Andrew Nowak, Alexander Senf, Jaime M Tovar-Corona, Alexander Vikhorev, Thomas M Keane, GA4GH Streaming Task Team, Dixie Baker, Vadim Zalunin, Angel Pizarro, Richard Durbin, Mark Diekhans, Edmon Begoli, Ilia Tulchinsky, Heng Li, Rishi Nag, Stephen Keenan, Ilkka Lappalainen, Jim Robinson

**Affiliations:** 1 Big Data Institute, Li Ka Shing Centre for Health Information and Discovery, University of Oxford, Oxford, UK; 2 DNAnexus, 1975 West El Camino Real, Suite 101, Mountain View, CA, USA; 3 Verily Life Sciences LLC, 269 East Grand Avenue, South San Francisco, CA, USA; 4 European Bioinformatics Institute, Wellcome Genome Campus, Hinxton, UK; 5 Wellcome Sanger Institute, Wellcome Genome Campus, Hinxton, UK; 6 Google Inc; 7 Wolfson Wohl Cancer Research Centre, Institute of Cancer Sciences, University of Glasgow, Glasgow, UK; 8 Global Alliance for Genomics and Health (ga4gh.org)

## Abstract

**Summary:**

Standardized interfaces for efficiently accessing high-throughput sequencing data are a fundamental requirement for large-scale genomic data sharing. We have developed htsget, a protocol for secure, efficient and reliable access to sequencing read and variation data. We demonstrate four independent client and server implementations, and the results of a comprehensive interoperability demonstration.

**Availability and implementation:**

http://samtools.github.io/hts-specs/htsget.html

**Supplementary information:**

Supplementary data are available at *Bioinformatics* online.

## 1 Introduction

We are witnessing a transition of sequencing technologies from being primarily a research platform to a routine clinical healthcare assay. In the next five years, it is predicted that the vast majority of the human genomes sequenced will be from healthcare (Birney *et al.*, 2017; https://doi.org/10.1101/203554). The sheer volume of human genetic data offers new opportunities for the discovery of new disease associations for common and rare disease. Most human genetic data is subject to participant consent agreements which define how the data can be shared. In research, The European Genome-phenome Archive (EGA), The database of Genotypes and Phenotypes (dbGaP) and the Japanese Genome-phenome Archive (JGA) have provided centralized repositories for sharing controlled access human data. As consented human data is increasingly subject to jurisdictional restrictions of data storage and rapidly increasing in scale, we are moving away from a few centralized repositories to a more distributed network of nodes (e.g. biobanks, national or regional healthcare providers, commercial clouds) housing large cohorts. In 2013, the Global Alliance for Genomics and Health was formed as a policy-framing and technical standards setting organization, akin to the W3C, to support the development of interoperable standards for discovering, sharing, accessing and storing genomic data (Birney *et al.*, 2017).

Standardization of next-generation sequencing file formats such as SAM/BAM for read data (Li *et al.*, 2009) and VCF/BCF for variants (Danecek *et al.*, 2011) was one of the key technical achievements from the 1000 Genomes Project. This has enabled a global ecosystem of interoperable sequence analysis tools, and production scale pipelines to be built around these formats. Sending genomic data over networks has largely been achieved by transporting files in these well-established formats using network protocols such as FTP/HTTP(s), Globus (Allcock *et al*. 2005; https://doi.org/10.1109/sc.2005.72) and proprietary protocols such as Aspera (http://asperasoft.com/). Commonly used tools such as Samtools (Li *et al.*, 2009), GATK (McKenna *et al.*, 2010), bedtools (Quinlan and Hall, 2010) and IGV (Robinson *et al.*, 2011) all support obtaining data over FTP/HTTP(s). Utilising open and widely implemented transfer protocols has ensured easy integration with common networking software libraries, reduced dependency on proprietary software or frameworks, and interoperability across network infrastructures.

This file-centric approach to defining and delivering data has substantial drawbacks, however, and there is an increasing consensus that more fine-grained and flexible data access methods are needed. Several such methods of storing and serving genomic data, such as GEMINI (Paila *et al.*, 2013), BGT (Li, 2016), GenAp (Kozanitis and Patterson, 2016) and Hail (Ganna *et al.*, 2016) have been developed. To avoid fragmentation and to promote a diverse ecosystem of interoperable storage and presentation technologies for genomic data, we need to develop standardized APIs for the discovery and delivery of read and variant data.

In this paper, we introduce a new open standard for real-time secure streaming of genomic data. This protocol, htsget, allows a client to retrieve data overlapping a specific genomic interval and uses existing community standards such as SAM/BAM/CRAM/VCF/BCF as the on-the-wire format. The protocol operates by a simple indirection layer, which provides many benefits over direct HTTP/FTP access to the data by clients. Secure access to data is supported by the industry-standard OAuth 2.0 protocol (https://tools.ietf.org/html/rfc6749).

The fundamental goal of htsget is to introduce a standardized interface for requesting and delivering genomic data that is not bound by file semantics. The protocol does not attempt to provide an end-to-end solution for managing genomic data. Issues around the organization of metadata and data discovery are outside the scope of this protocol. Future work will address these important issues; we envisage htsget as part of a family of loosely coupled protocols enabling efficient and secure discovery and exchange of genomic data.

## 2 Results

### 2.1 Schematic of protocol

**Fig. 1.**
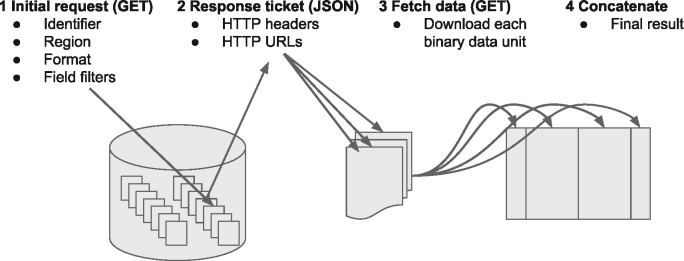
Schematic of htsget protocol

The key mechanic of the protocol is that the client provides a URL (determined via another discovery service), a preferred format and an optional genomic range via a HTTP(s) GET request (Fig. 1). The server returns a small JSON block with a list of URLs. The client downloads the data from the URLs, concatenates the downloaded data in the order provided by the server to produce the full result of their query.

### 2.2 Security

We envision that htsget will be used to stream both open genetic data and data that is subject to increased security and authorization requirements (e.g. consented human data). Sensitive information transmitted on public networks, such as access tokens and human genomic data, must be protected using Transport Level Security (TLS). Htsget can be integrated into existing authorization and authentication infrastructures (AAI) that use OAuth2.0 tokens to authorize data requests.

### 2.3 Initial implementations and interoperability

Htsget has been implemented by a set of research and commercial providers of human data, and community tool maintainers to develop a diverse set of client and server implementations. These include resources such as the European Genome-phenome Archive (EGA), Google Cloud Platform, SAMtools/HTSlib, DNAnexus and read browsers such as Integrated Genome Viewer (IGV) and Biodalliance (see Supplementary Material for full details).

To demonstrate interoperability, each server loaded the 1000 Genomes/Hapmap CEU trio in both BAM (NCBI37) and CRAM format (GRCh38), as well as RNASeq and ChIP-seq data. We developed a test application that runs a mixture of random and edge-case queries for a given client/server combination, checking the integrity of the returned data against a local file. We then used this application to run an extensive suite of tests on a total of 25 different client-server combinations. Full details are given in the supplementary Material.

## 3 Discussion

Files have long been the fundamental unit of bioinformatics, providing a simple abstraction for data organization and mapping naturally to Unix workflows. However, the explosive growth in the volume of genomics data and the increasingly distributed nature of computing resources are making this classical file-centric approach unsustainable. From a user perspective (i.e. the data consumer), downloading many terabytes of data simply to access a small subset of interest is clearly not satisfactory. Direct access to genomic loci in read or variant data over HTTP is a major improvement, but this is achieved by the means of auxiliary index files and requires that the server expose file semantics for all the data that it serves. This severely limits the flexibility of server providers in how data may be dynamically generated and organized, and also may require that several copies of near-identical data must be stored (if, for example, they wish to support different formats).

We have introduced htsget, an API for requesting and delivering genomic data. It builds on existing well-supported file formats for transport, while giving service providers freedom to use alternative internal data storage models. Using existing formats means that tools and data processing pipelines are only required to make minimal modifications to support htsget, exemplified by the list of initial implementations. There is a single layer of indirection which enables parallelism and robustness for service providers and clients. As htsget is intended to transport both open and controlled access data, appropriate security (TLS, HTTPS and OAuth2.0) are essential aspects of the specification.

## Supplementary Material

Supplementary Data
