# Supplementary material for: htsget: a protocol for securely streaming genomic data
Source: Bioinformatics. 2018 Jun 19;35(1):119–21. doi: 10.1093/bioinformatics/bty492 (PMC6298043; doi:10.1093/bioinformatics/bty492)
Supplement: Supplementary Data [file bty492_supplementary_material.pdf]

Implementations and Interoperability Testing

In this supplement we describe the diverse implementations of the htsget protocol that were developed, and the extensive testing performed to ensure that all implementations were fully interoperable.

Please see the htsget servers list for the endpoint URLS of servers hosting the interoperability test data.

1 Implementation details

In this section we provide some basic details about the various client and server implementations tested. This illustrates the wide range of different technologies, programming languages and platforms used.

1.1 DNAnexus

DNAnexus operates a demonstration htsget server, *htsnexus*, which serves access to BAM and CRAM files hosted in Microsoft Azure Blob Storage (ABS) and Amazon S3. The htsnexus server running under Node.js® consults a pre-loaded SQLite database with the cloud locations and block-by-block indices of each read group set.

Clients querying htsnexus receive back URLs and byte ranges on the cloud storage service directly, along with all necessary delegate credentials needed to access them. As a result, the htsnexus server only dispenses the small JSON signalling messages, while the massive read data payloads are transmitted to the client by ABS/S3 directly. This permits a small htsnexus server to handle requests from many parallel clients without becoming overloaded.

In addition to the server, the open source code includes a simple htsget client script written in Python.

URL: https://github.com/dnanexus-rnd/htsnexus
Contact: mlin@dnanexus.com

1.2 Google

The Google htsget server provides access to BAM (and, soon, CRAM) files stored in Google Cloud Storage (GCS). The htsget server can be deployed inside Google Cloud projects and read data from that project's (or any other project's) buckets with optional support for TLS and simple access control (for restricting access in non authenticated cases).

The server requires the target files to be indexed and the index files to be stored beside the primary data. The server reads the index to determine which chunks of the data file must be read and returned to the client.

The server is written in Go and is open source (Apache License).

URL: https://github.com/googlegenomics/htsget
Contact: kemp@google.com

1.3 Wellcome Sanger Institute NPG Ranger

Wellcome Sanger Institute provides, as one of its implementations of the htsget protocol, a JavaScript package with both server and client. They work on top of Node.js® runtime environment and rely on Node.js® streaming and process piping capabilities.

The server implementation is an abstraction layer which understands the protocol specification and provides the expected protocol endpoints using http/https. The abstraction layer decouples the data storage from the rest of the process, allowing for on-the-fly query region extraction and merging underlying distributed resources, transcoding between formats and limited capabilities for data integrity validation (through digest embedded in http trailers).

By design, authentication and authorization are abstracted and expected to be provided by external services. So far, the setup has been tested delegating authentication to Google Authentication service and authorization to a service which understands the institute's internal data access policies.

The client is provided as a CLI, but key parts of the protocol are implemented as a library which can be used independently. A proof of concept JavaScript genome browser was modified to display data by wrapping htsget requests using this library and browserify to transpile the Node.js JavaScript to a browser-friendly version.

URL: https://wtsi-npg.github.io/npg_ranger/
Contact: david.jackson@sanger.ac.uk

1.4 EGA

The European Genome-phenome Archive (EGA) is a service and database for permanent archiving and sharing of all types of personally potentially identifiable genetic and phenotypic human data resulting from biomedical research projects. In 2018, the EGA data access API (v3) has added support for htsget. The archive and permissions structure of EGA necessitates that IDs used are EGA File IDs, and the API requires a valid EGA or ELIXIR AAI JWT to provide access.

The htsget 1.0 compliant API is receives the request for BAM/CRAM files on /files/{id} and for VCF/BCF Files on /variants/{id}, and returns a JSON response ticket containing the URL(s) where the data can be downloaded. The Data/Blob server is part of the same microservice.

The server is written in Java 1.8 and relies on htsjdk. All code is open source (Apache 2).

URL: https://ega.ebi.ac.uk:8051/elixir/data/tickets
GitHub: https://github.com/EGA-archive/ega-dataedge
Contact: asenf@ebi.ac.uk

1.5 Htslib

HTSlib is a C library which allows applications to read and write sequence alignments in SAM, BAM and CRAM formats, and variant data in VCF and BCF2 files. It can access files both on local storage and over the network using via FTP, HTTP and HTTPS (if linked with the libcurl library). HTSlib includes file type detection so that reading different formats is, as far as possible, transparent to the end user.

When it opens an htsget URL, HTSlib detects and parses the htsget JSON response. It then downloads each chunk in sequence and passes the data back through the original file handle. From the point of view of the reader, this makes htsget look like any other streamed alignment file. This means most tools that use HTSlib for file access can read data via htsget by simply specifying the htsget URL as the location of the input file.

Support for the current htsget specification was added in HTSlib release 1.6. Release 1.7 added basic support for sending Oauth2 bearer tokens. Support for htsget requires HTSlib to be built against libcurl.

URL: https://github.com/samtools/htslib/
Contact: samtools-devel@lists.sourceforge.net

1.6 Htsget

Htsget is a Python client implementation of the htsget protocol (the software was named before the protocol adopted the same name). It is based on the requests library, and is tested on the Linux, Mac and Windows operating systems. Htsget provides a simple Python API as well as a command line interface for the protocol, and can be installed from PyPI.

The library provides reliable access to data by retrying any chunks that fail to download a configurable number of times, thus working around transient network issues. Download performance is excellent, providing download speeds indistinguishable from using cURL or wget.

Please see the documentation for more details.

URL: https://pypi.python.org/pypi/htsget
Contact: jerome.kelleher@well.ox.ac.uk

2 Testing and interoperability application

We developed an application to test the data integrity of client and server implementations. The tester is provided a local copy of a BAM or CRAM file and a URL for a htsget endpoint representing the same data. The tester operates by running a set of queries on the local and remote version of the data, and then verifies that data returned is equivalent to the local version. The tester is written in Python, and uses the pysam library for reading BAM and CRAM files.

For a given client and server combination, the tester works by examining the local data, and constructing a series of queries. For a given query, the tester calls the client under test with the server URL and query parameters as arguments, and write the results to a temporary file. This temporary file is then read, and compared to the data from the local source file (which should be identical to the data stored on the server). Any data mismatches are reported, and summarised at the end of a test run.

The queries run are a mixture of edge-cases overlapping the ends of contigs in various ways, full contig fetches, and a configurable number of randomly generated start and end coordinates.

The code is available on GitHub.

3 Test data

The data used for the interoperability tests was a mixture of CRAM and BAM data from the 1000 Genomes CEU trio (NA12878, NA12891 and NA12892) mapped to different reference versions. RNASeq and Chip-seq BAMs were also included.

Description	Assembly	File	Type
CEU Daughter	GRCh37	NA12878.bai	HiSeqX BAM index
		NA12878.bam	HiSeqX BAM
CEU Father	GRCh37	NA12891.bai	HiSeqX BAM index
		NA12891.bam	HiSeqX BAM
CEU Mother	GRCh37	NA12892.bai	HiSeqX BAM index
		NA12892.bam	HiSeqX BAM
CEU Daughter	GRCh38	NA12878.crai	HiSeqX CRAM index
		NA12878.cram	HiSeqX CRAM
CEU Father	GRCh38	NA12891.crai	HiSeqX CRAM index

		NA12891.cram	HiSeqX CRAM
CEU Mother	GRCh38	NA12892.crai	HiSeqX CRAM index
		NA12892.cram	HiSeqX CRAM
GM12878 immortalized cell line of NA12878	GRCh38	ENCFF284YOU.bam	RNAseq BAM
GM12878 immortalized cell line of NA12878	GRCh37	ENCFF000VWO.bam	ChIP-seq BAM

4 Interoperability results

	**Servers**									
	**WSI**		**DAWS**		**DAZ**		**GCP**		**EGA**	
**Clients**	BAM	CRAM	BAM	CRAM	BAM	CRAM	BAM	CRAM	BAM	CRAM
htsget								n/a		n/a
WSI								n/a		n/a
EGA								n/a		n/a
Samtools								n/a		n/a

Table 1: Interoperability status for clients (side) and servers (top). Green boxes indicate fully passing interoperability (see 2 above for details), yellow indicates combination not supported by the server. WSI=Wellcome Sanger Institute, DAWS=DNAnexus on Amazon AWS, DAZ=DNAnexus on Azure, GCP=Google Cloud Platform, EGA=European Genome-phenome Archive. Server URLs are given in section 1 above.
